# RETRACTION: Interleukin 10 Plays an Important Role in Neonatal Rats with Hypoxic-Ischemia Associated with B-Cell Lymphoma 2 and Endoplasmic Reticulum Protein 29

**DOI:** 10.1155/ancp/9867036

**Published:** 2025-09-10

**Authors:** Analytical Cellular Pathology

RETRACTION: X. Bai, L-L. Xiong, C-L. Fang, et al., “Interleukin 10 Plays an Important Role in Neonatal Rats with Hypoxic-Ischemia Associated with B-Cell Lymphoma 2 and Endoplasmic Reticulum Protein 29,” *Analytical Cellular Pathology* 2021 (2021): 6622713, https://doi.org/10.1155/2021/6622713.

The above article, published online on 02 June 2021 in Wiley Online Library (wileyonlinelibrary.com), has been retracted by agreement between the journal Editor-in-Chief, Dimitrios Karamichos, and John Wiley & Sons Ltd. The retraction has been agreed due to concerns about the reliability of the Western Blots in Figure 2a, c, d following an initial request from the authors to make corrections.

The authors initially requested to:

• Replace the ß-actin bands in Figure 2a, as they feel that the 24 h band is weaker than the others.
• Replace the IL-10 band in Figure 2a to keep the ratio, maintaining the same histogram.
• Replace ß-actin bands in Figure 2d due to inconsistencies in the size and shape of the bands over the different time periods.

Subsequent investigation by the journal identified that the histograms in Figure 2c,d are also identical.

The editorial board has determined that the data in the article is unreliable due to concerns over the accuracy of the blots and histograms in Figure 2, as well as the issue of figure duplication. As a result, the article is retracted.

The authors agree to the retraction.

